# New concepts in breast cancer genomics and genetics

**DOI:** 10.1186/s13058-014-0460-4

**Published:** 2014-10-23

**Authors:** Rodrigo Goncalves, Wayne A Warner, Jingqin Luo, Matthew J Ellis

**Affiliations:** 1 Breast Cancer Program, Department of Medical Oncology, Washington University School of Medicine, 660 S. Euclid Ave, St Louis, 63110 MO USA; 2 Siteman Cancer Center, Washington University School of Medicine, 660 S. Euclid Ave, St Louis, 63110 MO USA; 3 Department of Cell Biology and Physiology, Washington University, 660 S. Euclid Ave, St Louis, 63110 MO USA; 4 Division of Biostatistics, Washington University School of Medicine, 660 S. Euclid Ave, St Louis, 63110 MO USA; 5 Lester and Sue Smith Breast Center, Baylor College of Medicine, One Baylor Plaza, 320A Cullen MS600, Houston, 77030 TX USA

## Abstract

Massively parallel DNA and RNA sequencing approaches have generated data on thousands of breast cancer genomes. In this review, we consider progress largely from the perspective of new concepts and hypotheses raised so far. These include challenges to the multistep model of breast carcinogenesis and the discovery of new defects in DNA repair through sequence analysis. Issues for functional genomics include the development of strategies to differentiate between mutations that are likely to drive carcinogenesis and bystander background mutations, as well as the importance of mechanistic studies that examine the role of mutations in genes with roles in splicing, histone methylation, and long non-coding RNA function. The application of genome-annotated patient-derived breast cancer xenografts as a potentially more reliable preclinical model is also discussed. Finally, we address the challenge of extracting medical value from genomic data. A weakness of many datasets is inadequate clinical annotation, which hampers the establishment of links between the mutation spectra and the efficacy of drugs or disease phenotypes. Tools such as dGene and the DGIdb are being developed to identify possible druggable mutations, but these programs are a work in progress since extensive molecular pharmacology is required to develop successful ‘genome-forward’ clinical trials. Examples are emerging, however, including targeting HER2 in HER2 mutant breast cancer and mutant ESR1 in *ESR1* endocrine refractory luminal-type breast cancer. Finally, the integration of DNA- and RNA-based sequencing studies with mass spectrometry-based peptide sequencing and an unbiased determination of post-translational modifications promises a more complete view of the biochemistry of breast cancer cells and points toward a new discovery horizon in our understanding of the pathophysiology of this complex disease.

**Electronic supplementary material:**

The online version of this article (doi:10.1186/s13058-014-0460-4) contains supplementary material, which is available to authorized users.

## Introduction

A decade after the first version of the human genome was published [1], annotation efforts continue, bringing us to the 19th revision, which is the current research standard. Analysis of protein-coding genes and their regulatory sequences is nearing completion, but these functions are served by only a small fraction of the genome. The rest is more functional than once thought, encoding, for example, many non-protein coding RNA genes with emerging regulatory and catalytic roles in cellular physiology and cancer [2]. Furthermore, mass spectrometry-based peptide sequencing is rapidly maturing, promoting studies that provide an unbiased analysis of information flowing from DNA to mRNA to protein to post-translational modification without the need for probes or antibodies at the individual gene or protein level [3]. Finally, deregulation of histone function and DNA methylation is readily evident in many tumor types and is a further consideration in cancer pathogenesis [4]. There is a growing chasm between our understanding of the breast cancer genome and our ability to translate these insights into improved patient outcomes. In this review, we present some of the most recent findings in the genomics field, from the biological discoveries emanating from genome sequencing studies to the clinical implications of those findings and finally to the future areas of potential research in the field.

## Recent biologically relevant findings in the genomics field

### Significantly mutated genes versus background mutations in breast cancer

Sequencing of DNA and RNA from tumors by using massively parallel sequencing with a capture or other sequence selection approach (exomes or candidate genes) or unbiased ‘whole genome’ approach has become a standard research tool now that the technology has been extensively commercialized [5]-[7]. One objective of cancer sequencing studies is to identify genes that have undergone somatic mutations, which contribute to malignant transformation. Genes that accumulate somatic mutations at a higher than stochastic rate are referred to as ‘significantly mutated genes’ (SMGs) and are considered likely drivers of malignant progression. In breast cancer, there is a dramatic difference in the SMG list between luminal-type breast cancer and basal-like breast cancer. In The Cancer Genome Atlas (TCGA) breast cancer data, at least 20 SMGs were observed in luminal-type A, eight in luminal-type B, but only three in basal-like breast cancer (Table 1). This is not because luminal breast cancer genomes are more complex than those of basal-like breast cancer; in fact, the opposite is true. Basal-like breast cancer genomes are often so complex that it has proven difficult to identify the causal events by using mutation recurrence statistics. Furthermore, structural rearrangements (large-scale chromosomal deletions, amplifications, inversions, and translocations) are likely to play a particularly critical role in basal-like breast cancer, and the complete delineation of these events requires whole genome sequencing, which is technically demanding and expensive [8].

**Table 1:** **Significantly mutated genes based on all luminal versus basal-like breast cancers in The Cancer Genome Atlas dataset**

Gene	Luminal A (n = 225)			Luminal B (n = 126)			Basal-like (n = 93)		
	Number of cases	LRT	CT	Number of cases	LRT	CT	Number of cases	LRT	CT
*TP53*	28	0	0	39	0	0	74	0	0
*PIK3CA*	105	0	0	40	0	0	8	4.0 × 10^−6^	3.4 × 10^−7^
*GATA3*	32	0	0	19	0	0	2	NA	NA
*MAP3K1*	30	0	0	6	1.7 × 10^−8^	4.7 × 10^−7^	0	NA	NA
*MLL3*	19	1.5 × 10^−10^	1.7 × 10^−11^	7	NA	NA	6	NA	NA
*CDH1*	23	0	0	6	3.6 × 10^−3^	6.6 × 10^−3^	0	NA	NA
*MAP2K4*	16	0	0	3	NA	NA	0	NA	NA
*RUNX1*	13	0	0	3	NA	NA	0	NA	NA
*PTEN*	9	4.3 × 10^−9^	1.3 × 10^−11^	6	3.7 × 10^−6^	1.9 × 10^−7^	1	NA	NA
*TBX3*	6	1.0 × 10^−6^	2.7 × 10^−5^	6	9.4 × 10^−5^	1.4× 10^−4^	1	NA	NA
*PIK3R1*	4	NA	NA	4	NA	NA	2	NA	NA
*AKT1*	8	1.4× 10^−11^	3.2× 10^−9^	3	NA	NA	0	NA	NA
*CBFB*	5	4.2× 10^−5^	2.7× 10^−5^	2	NA	NA	0	NA	NA
*TBL1XR1*	5	2.9× 10^−2^	1.2× 10^−3^	1	NA	NA	0	NA	NA
*NCOR1*	12	3.8× 10^−8^	6.8× 10^−9^	3	NA	NA	2	NA	NA
*CTCF*	9	8.8× 10^−4^	3.0× 10^−6^	2	NA	NA	1	NA	NA
*ZFP36L1*	2	NA	NA	4	1.3× 10^−2^	1.7× 10^−2^	1	NA	NA
*GPS2*	4	1.2× 10^−3^	6.0× 10^−3^	1	NA	NA	1	NA	NA
*SF3B1*	7	1.1× 10^−6^	5.3× 10^−5^	0	NA	NA	1	NA	NA
*CDKN1B*	3	5.4× 10^−3^	1.9× 10^−2^	1	NA	NA	0	NA	NA
*USH2A*	7	NA	NA	4	NA	NA	10	NA	NA
*RPGR*	2	NA	NA	2	NA	NA	4	NA	NA
*RB1*	1	NA	NA	4	NA	NA	4	2.5× 10^−2^	4.8× 10^−2^
*AFF2*	3	NA	NA	3	NA	NA	4	NA	NA
*NF1*	6	NA	NA	5	NA	NA	2	NA	NA
*PTPN22*	1	NA	NA	3	NA	NA	0	NA	NA
*RYR2*	6	NA	NA	10	NA	NA	2	NA	NA
*PTPRD*	4	NA	NA	5	NA	NA	1	NA	NA
*OR6A2*	2	NA	NA	1	NA	NA	0	NA	NA
*HIST1H2BC*	1	NA	NA	1	NA	NA	1	NA	NA
*GPR32*	3	8.9× 10^−3^	4.3× 10^−2^	1	NA	NA	1	NA	NA
*CLEC19A*	0	NA	NA	1	NA	NA	0	NA	NA
*CCND3*	2	1.5× 10^−4^	1.1× 10^−3^	0	NA	NA	0	NA	NA
*SEPT13*	2	NA	NA	0	NA	NA	1	NA	NA
*DCAF4L2*	1	NA	NA	3	NA	NA	1	NA	NA

CT, chemotherapy; LRT, loco-regional treatment; NA, mutations observed were not considered statistically significant.

Detection of SMGs is complicated by the presence of a large number of likely irrelevant mutations referred to as ‘background mutations’ [9]-[11]. These occur not only in genes irrelevant to transformation but even within the SMGs themselves; that is, a missense mutation in a large tumor suppressor gene cannot be assumed to be always inactivating or cause dysfunction in the encoded protein. Mutant allele expression determined by RNA sequencing (RNA seq) is one starting point for disambiguating biologically relevant mutations on SMGs versus irrelevant ones. Many mutations detected at the DNA level are not expressed at the RNA level and thus, at least from the gain-of-function perspective, are unlikely to be major players in the carcinogenesis process [12]. Although there are challenges left to functionalize many of the SMGs as drivers of carcinogenesis, some progress has been made. RNA seq is widely used for the nomination and validation of expressed fusion genes and was recently used to define an endocrine therapy resistance-associated ESR1 translocation [12]. Ultimately, functional studies are critical for resolving the role of mutations in certain SMGs versus background mutations, since the large number of mutations requiring annotation creates an extreme challenge, if this is done in an unbiased way [13]. An alternative approach is to be selective and initially study those associated with a therapeutic hypothesis. Another priority consists of the SMGs themselves, as the biology served by many of these, particularly those involved in mechanisms such as histone methylation, splicing, transcription, and long non-coding (*lnc*) RNA function is unclear. For example, whole genome analysis revealed clustered mutations in *MALAT1*, suggesting a gain-of-function role for this poorly understood and abundant *lnc*RNA in breast cancer [14]. The functions of luminal SMGs have particularly striking similarities to drivers in hematopoietic malignancies [14], a link also emphasized by a recent study on the role of estradiol in hematopoiesis [15]. A particularly vexing problem is the functional resolution of mutated genes that drive pathogenesis in just a few patients or even in only one patient. A significant number of cases of luminal-type breast cancer in the TCGA analysis did not harbor a single SMG [16], suggesting that current genomic approaches would potentially benefit from additional refinement.

### The genomic structure of breast cancer reveals underlying DNA repair defects

**Figure 1:**
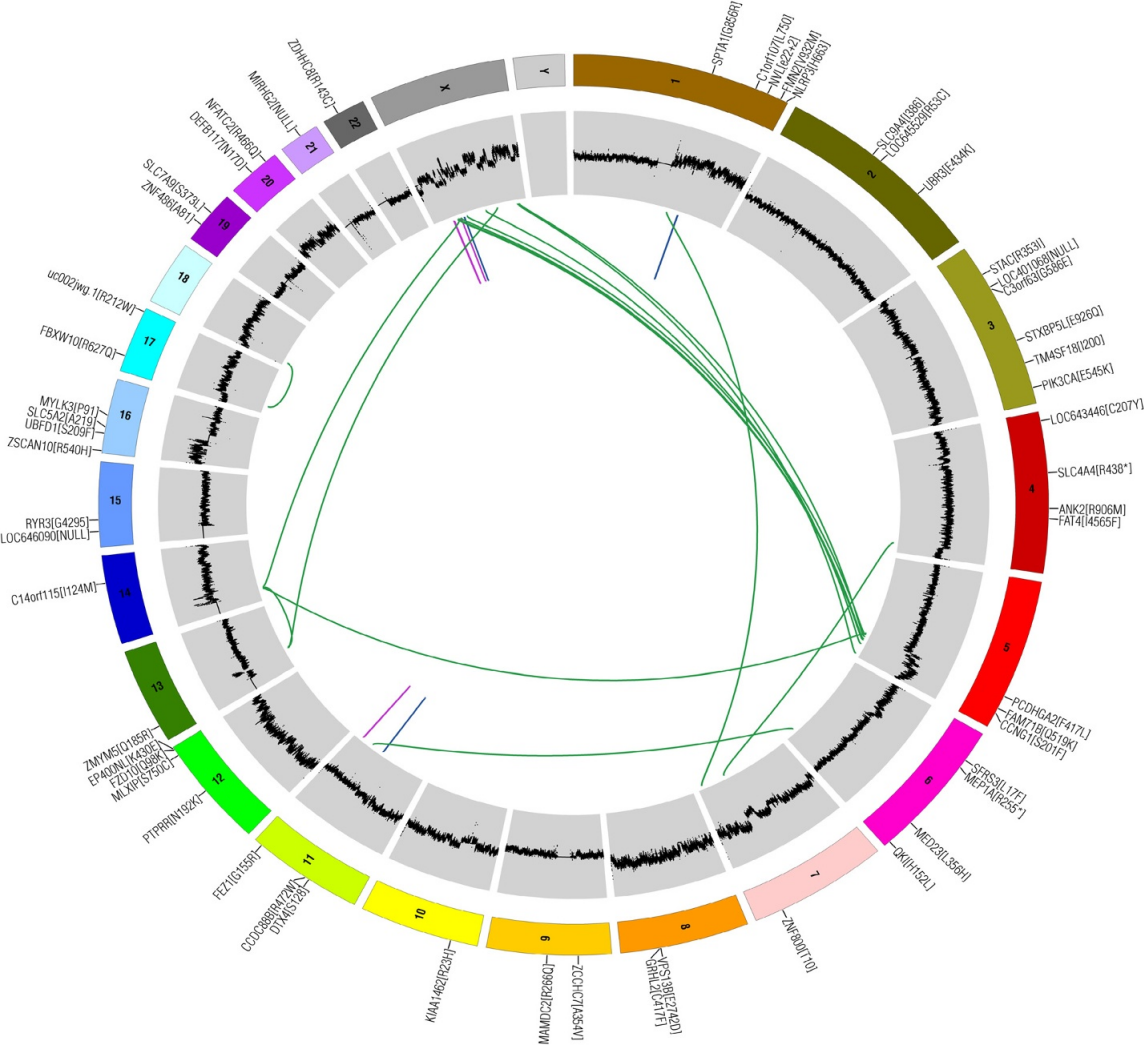
**The presence of translocations and amplification at the ends of the breakpoints is evidence of chromothripsis in this Circos plot from a breast cancer sample.** Chromothripsis scars the genome when localized chromosome shattering and repair occur in a one-off catastrophe.

Aside from the focus on the identification of individual genes that are repetitively disrupted in breast cancer, a more broad-based analysis of breast cancer genome structures has led to a paradigm shift in the way we view pathogenesis. The standard multistep model of carcinogenesis postulates that mutations accumulate gradually, one at a time, in a process of Darwinian selection in which individual mutant-bearing clones effectively compete with normal cells and other clones within the tumor through the acquisition of the ability to transform, invade, metastasize, and evade drug treatment [17]. However, it was recently demonstrated that multiple mutations can arise over a very short period wherein multiple chromosomal breaks that occurred during a single catastrophic cell division event are (rarely) viably repaired, reshuffling the genome in a way that rapidly triggers transformation though the simultaneous oncogene amplifications and tumor suppressor gene deletions in the vicinity of the multiple translocations that ensue (chromothripsis) [18] (Figure 1). The reported frequency of chromothripsis in breast cancer varies from 2% to 11.06% [18],[19]. Since chromothripsis and interval breast cancer are both marked by the suddenness of their appearance, we hypothesize that chromothripsis might explain the development of rapidly progressing, so-called ‘interval’, breast cancers that arise suddenly between screening visits. For this class of tumors, screening could never be effective as the time span of tumor development is too short. The genomic structure of interval breast cancers should be pursued aggressively as these tumors carry a high mortality burden. As more patients are included in clinical trials that include longitudinal genome sequencing of tumor samples, this hypothesis will be tested in the near future.

In another conceptual breakthrough, investigators at the Sanger Institute demonstrated that there are more than 20 different patterns of somatic mutation in cancer based on copy number aberrations and nucleotide substitution patterns, with a subset of these recurrently observed in breast cancer (APOBEC, BRCA1/2, Signature B) [20]. Overexpression of cytidine deaminase APOBEC family members, in particular, has come into sharp focus. Clustered mutations characteristic of APOBEC activity have been particularly observed in and around chromosomal breakpoints, suggesting that single-stranded DNA generated during aberrant DNA repair is a substrate for APOBEC enzymatic activity [21]. Differences in DNA repair defects explain the striking finding that some breast cancers display many more mutations than others [20],[22]. Thus, even in the absence of a known SMG, it is possible to classify breast cancers on the basis of DNA repair defects and this could be clinically relevant. For example, clinical assays in development aim to identify tumors with defects in homologous recombination, which sensitize tumors to cytotoxic chemotherapy [23].

### Intra-tumor heterogeneity in breast cancer

Chromothripsis, multistep progression, and defects in DNA repair combine to produce astonishing levels of both intra-tumoral and inter-tumoral heterogeneity in breast cancer. This complexity is an obvious explanation for the difficulty in curing breast cancer, particularly when advanced. As the tumor progresses and disseminates, the repertoire of biological possibilities encoded within billions of malignant cells, each subtly genetically different, means that resistance to targeted or more traditional cytotoxic therapy is almost inevitable. There is still not enough genomic data from multiple cancer samples from the same patient to track somatic mutation patterns from the primary through to metastatic disease and subsequent drug resistance. Longitudinal studies of this type, however, have been conducted successfully in individual cases. In 2009, Shah and colleagues [24] described the mutational evolution of a lobular breast carcinoma by using next-generation sequencing. Out of the 32 somatic, protein-coding mutations present in the metastasis, 19 could not be detected in the primary, five were prevalent in the primary, and six were present in the primary with a lower frequency. The Washington University group investigated the progression of a breast cancer to the brain at the whole genome level and found that the primary tumor and metastasis harbored approximately 48 somatic, protein-coding mutations [8]. In the metastatic sample, there were few *de novo* mutations, but higher variant allele frequencies and a few much lower, supporting a ‘clonal remodeling’ hypothesis for metastatic spread. At the single cell level of the tumor, various techniques have been used to directly visualize and quantify chromosomal aberrations, including duplications, deletions, and other distinctive chromosomal rearrangements. These studies show that breast cancers routinely exhibit genetic heterogeneity at preferred loci [25]-[29].

Evidence for marked tumor heterogeneity can be found in studies of other cancer types. For example, in a study of a renal cancer with metastasis to the lung and in the chest wall, sequencing of the metastases and nine different areas within the primary tumor found that only a third of mutations were common to all samples [30]. Based on these data, we can infer that heterogeneity and different subclones develop within the primary tumor, not all of which have the same metastatic potential. Metastases can develop early or late in each cancer’s evolutionary history and are products of ongoing clonal evolution, which can be slow or very rapid. The ability to sequence individual cancer cells [31] will further illuminate this issue, although the complexity of the data analysis remains a considerable challenge.

## Clinical implications of genomic discoveries

### Clinical translation of massively parallel sequencing of DNA in breast cancer

The sequencing of cancer with data return to the patient and physician is being piloted through ‘genomic tumor boards’ [32]. However, the complexity of the breast cancer genome has slowed progress, as has the relative paucity of obvious drug mutation matches [33]. Unlike drug therapy matched somatic mutations to melanoma and non-small cell lung cancer, drug therapy matched to the presence of a somatic mutation has yet to be robustly established as a standard approach in breast cancer. A number of strategies to increase the productivity and ‘translatability’ of DNA, RNA, and peptide sequencing studies in breast cancer should be considered. The initial set of sequencing-based studies in breast cancer revealed that this is one of the most heterogeneous forms of cancer, with the four commonly accepted subtypes (luminal-type A, luminal-type B, HER2-enriched, and basal-like) displaying distinct somatic mutation, gene copy, and epigenetic profiles [16]. Within the next few years, tens of thousands of primary breast cancers will likely be sequenced but often through clinical sequencing programs without a current systematic and broad-based plan to integrate the data with clinical endpoints. These studies risk following the course of the TCGA breast cancer study. While a technical tour de force, TCGA was largely a cross-platform genome-cataloging exercise and not a systematic clinical research addressing a particular problem in oncology [16]. Thus, it will not be possible to link the TCGA data to important clinical phenotypes such as drug response. Since polypharmacy is the rule in breast cancer treatment, establishing a link between mutational events and the efficacy of individual drugs is impossible unless a dedicated study is conducted. The neoadjuvant treatment setting allows ethical treatment plans with single agents as well as the acquisition of serial samples to assess the effect of treatment on breast cancer somatic genomes - another subject in its infancy in breast cancer. Thus, a systematic approach linking high-quality sample acquisition, uniform neoadjuvant therapy regimens, and integrated ‘omics’ should be a high priority for clinical investigators. An example is provided by an integrated analysis of whole genome, exome-based somatic mutation detection, gene-expression, and gene copy profiles that identified molecular correlates of aromatase inhibitor-resistant proliferation by using samples from a neoadjuvant study [14]. Mutations in *TP53* were associated with endocrine therapy resistance, poor prognosis luminal-type B features, mutations in the stress kinase *MAP3K1* with low proliferation and luminal-type A features, and mutations in *GATA3* with increased responsiveness to aromatase inhibition. A current research focus is to confirm these findings and to conduct additional studies with large sample sizes to link other breast cancer SMGs to clinical outcomes.

### The druggable breast cancer genome

A major obstacle to the translation of newly defined genetic alterations into clinical benefit for patients lies in the identification of biologically relevant druggable aberrations that can be used as therapeutic targets [34]. To address this goal, programs such as dGene [35] and DGIdb [36] have been developed. The dGene program is an updated version of the druggable genome concept introduced in 2002 by Hopkins and Groom [37]. The druggable genome refers to a subset of genes that are known or predicted to interact with drugs. The software stratifies mutations from any database containing gene symbols into 10 different gene classes that are both potentially druggable and clinically relevant to cancer biology. An annotation and filtering tool is used to prioritize mutations for consideration. The analysis of a recent breast cancer genomic study [14] highlights the potential utility of this approach. From a total of 2,622 single-nucleotide variants identified in the neoadjuvant aromatase inhibitor discussed above, dGene identified 368 mutations out of 2,622 single-nucleotide variants as occurring in 255 druggable genes. When filtered for recurrence, that number was narrowed to 37 potentially druggable mutated genes present in at least two patients (Table 2). Despite its utility, dGene does not provide information on the type of mutation or guarantee clinical pertinence of mutations associated with any specific gene. This underscores the critical need to functionally test these and other genomic results.

**Table 2:** **Categorization of single-nucleotide variants in 77 breast cancer tumors using dGene: 37 dGene entries present in at least 2 out of 77 samples, organized by class and patients affected**

NCBI symbol	Full name	dGene class	Patients affected
CASR	Calcium-sensing receptor	G protein-coupled receptor	3
GPR112	G protein-coupled receptor 112	G protein-coupled receptor	3
AGTR2	Angiotensin II receptor, type 2	G protein-coupled receptor	2
MC5R	Melanocortin 5 receptor	G protein-coupled receptor	2
OR2L2	Olfactory receptor, family 2, subfamily L, member 2	G protein-coupled receptor	2
OR51B5	Olfactory receptor, family 51, subfamily B, member 5	G protein-coupled receptor	2
PIK3CA	Phosphoinositide-3-kinase, catalytic, alpha polypeptide	PI3K	37
BIRC6	Baculoviral IAP repeat containing 6	Proteinase inhibitor	4
CPAMD8	C3 and PZP-like, α-2-macroglobulin domain containing 8	Proteinase inhibitor	3
COL28A1	Collagen, type XXVIII, alpha 1	Proteinase inhibitor	2
COL6A3	Collagen, type VI, alpha 3	Proteinase inhibitor	2
AGBL1	ATP/GTP binding protein-like 1	Protease	2
CPVL	Carboxypeptidase, vitellogenic-like	Protease	2
PCSK5	Proprotein convertase subtilisin/kexin type 5	Protease	2
RELN	Reelin	Protease	2
SENP7	SUMO1/sentrin specific peptidase 7	Protease	2
USP9X	Ubiquitin specific peptidase 9, X-linked	Protease	2
PTPRF	Protein tyrosine phosphatase, receptor type, F	Phosphatase	2
PTPRU	Protein tyrosine phosphatase, receptor type, U	Phosphatase	2
SSH3	Slingshot homolog 3 (Drosophila)	Phosohatase	2
MAP3K1	Mitogen-activated protein kinase kinase kinase 1	Serine theonine kinase	9
TTN	Titin	Serine theonine kinase	6
ATR	Ataxia telangiectasia and Rad3 related	Serine theonine kinase	5
OBSCN	Obscurin	Serine theonine kinase	3
SMG1	Smg-1 homolog	Serine theonine kinase	3
ALPK2	Alpha-kinase 2	Serine theonine kinase	2
BRAF	V-raf murine sarcoma viral oncogene homolog B1	Serine theonine kinase	2
DCLK3	Doublecortin-like kinase 3	Serine theonine kinase	2
LRRK2	Leucine-rich repeat kinase 2	Serine theonine kinase	2
MAP2K4	Mitogen-activated protein kinase kinase 4	Serine theonine kinase	2
TAF1L	TATA box binding protein (TBP)-associated factor	Serine theonine kinase	2
TBK1	TANK-binding kinase 1	Serine theonine kinase	2
ULK4	Unc-51-like kinase 4	Serine theonine kinase	2
INSRR	Insulin receptor-related receptor	Tyrosine kinase	3
KIT	C-kit	Tyrosine kinase	2
PDGFRA	Platelet-derived growth factor receptor	Tyrosine kinase	2
TEX14	Testis expressed 14	Tyrosine kinase	2

NCBI, National Center for Biotechnology Information.

**Figure 2:**
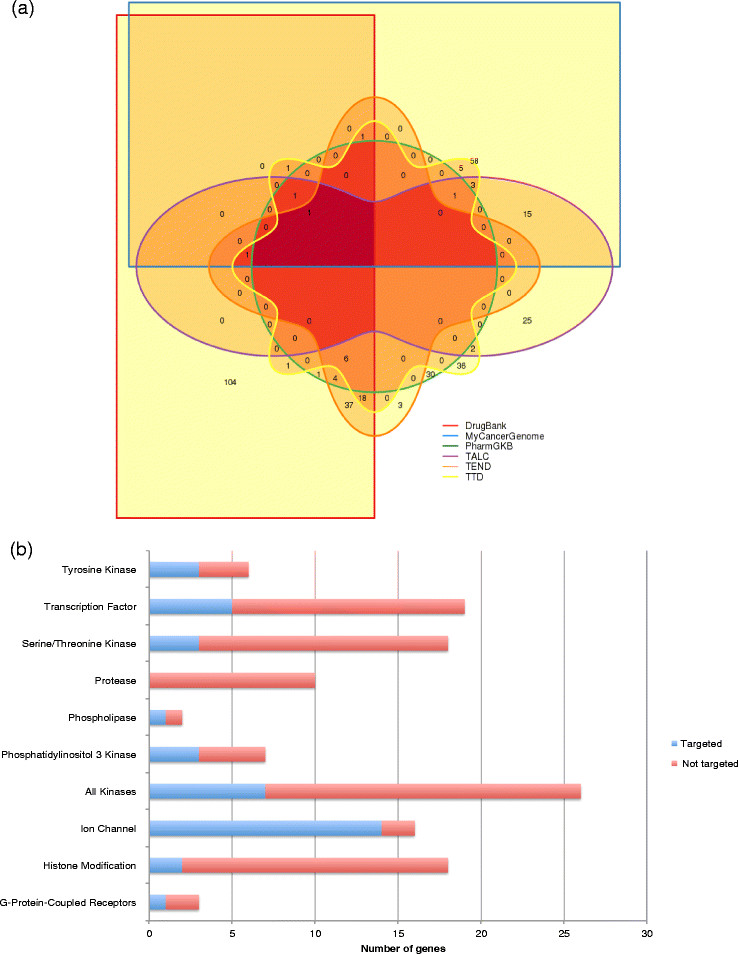
**Druggability of significantly mutated gene (SMG) in breast cancer. (a)** Overlap between six sources that generated a list of 354 possible drug-gene interactions among 315 genes recurrently mutated in breast cancer patients and analyzed by DGIdb. One hundred and seventy-six drug-gene interactions were identified by DrugBank, 87 by MyCancerGenome, 77 by Therapeutic Target Database (TTD), 71 by Trends in the Exploitation of Novel Drug Targets (TEND), 49 by Targeted Agents in Lung Cancer (TALC), and 44 by the Pharmacogenetics Knowledge Base (PharmGKB). **(b)** Distribution of 315 genes in potentially druggable categories (from dGene) and the numbers of genes in these categories that are targeted by a known drug.

A similar tool is DGIdb [36]. The concept behind the DGIdb is to classify gene mutations into two classes: genes that are known to have drug interactions and genes that are potentially druggable according to their gene category. DGIdb was developed by integrating data from 13 different sources and contains over 14,000 drug-gene interactions. It also includes 6,761 genes that belong to one or more of 39 potentially druggable gene categories. The utility of DGIdb was demonstrated by analyzing a cohort of 1,273 patients who were included in whole-genome or exome sequencing studies [16],[38]-[41]. The software identified 6 of 31 genes (*AKT1*, *CDH1*, *LRP2*, *PIK3CA*, *RYR2*, and *TP53*) that were recurrently mutated in at least 2.5% of patients and also have known drug-gene interactions. With the addition of the top 1% of recurring mutations, the number of genes increased to 315. Six sources - DrugBank, MyCancerGenome, the Pharmacogenetics Knowledge Base (PharmGKB), Trends in the Exploitation of Novel Drug Targets (TEND), Targeted Agents in Lung Cancer (TALC), and Therapeutic Target Database (TTD) - were interrogated by DGldb to identify a total of 354 possible druggable gene interactions among the 315 genes. There was limited overlap between the sources, and only one drug-gene interaction was present in all six sources simultaneously (Figure 2a). The nature and extent of curation as well as the overall methodologies employed by each source are different (Figure 2a), which explains the limited overlap between the different sources. Some of the 315 genes are in potentially druggable categories (dGene), and others represent opportunities for drug discovery (Figure 2b).

This analysis serves to emphasize that these druggable genome approaches remain unvalidated by clinical trials and the pre-existing pharmacopeia is obviously inadequate, although ‘drug repurposing’ - the concept of redirecting US Food and Drug Administration-approved drugs to new secondary indications - is clearly an opportunity. Thus, in their current form, these computational approaches are mostly hypothesis-generating tools that are intended to accelerate medical research, not tools for clinical action (at least not yet). The next logical step after using such tools is to design functional studies to test the related drugs and find a more reliable answer as to whether such mutations are drivers of carcinogenesis or just background mutations.

### HER2 and ESR1 mutations as examples of novel druggable targets

The utility of detailed preclinical work on potentially druggable genes is nicely illustrated by the study of *HER2* mutations in breast cancer. Data from eight breast cancer genome-sequencing studies identified 25 patients with *HER2* somatic mutations without *HER2* amplification [14],[16],[24],[38]-[42]. Thirteen *HER2* mutations were functionally characterized by using *in vitro* kinase assays, protein structure analysis, cell culture, and xenograft experiments [43]. The results showed that the investigational drug neratinib, an irreversible *HER2* inhibitor, rather than lapatinib, an approved *HER2* kinase inhibitor, was a better approach for clinical studies since some of the recurrent mutations were naturally lapatinib-resistant. This is a result that simple drug somatic mutation matching software would not have revealed. Currently, patients with advanced *HER2* mutation-positive tumors are being enrolled into a single-agent study of neratinib (NCT01670877). Point mutations in the estradiol-binding domain of the estrogen receptor gene (ESR1) are emerging as a potent cause of acquired endocrine therapy resistance. Although there are no drugs that specifically target these mutations, alternative endocrine therapies may be effective in this setting [44],[45] and this possibility will soon be addressed in clinical trials.

### Patient-derived xenografts as genomic models for breast cancer

A major criticism of standard cell lines as a model for human breast cancer is that they are essentially disconnected from the individuals from whom they were derived. Without knowledge of the progenitor tumor genome as a reference point and no knowledge of the clinical characteristics of the patient who donated the tissue, it is uncertain what the cell lines actually model from an individual patient perspective and to what degree genetic drift has occurred after prolonged *in vitro* culture. These limitations likely contribute to the poor predictive utility of cell line panels in drug development [46]-[48]. An alternative preclinical model for drug optimization and target validation is the patient-derived xenograft (PDX) approach. Detailed information covering the continuum from specimen acquisition to development of patient-derived xenografts has been presented and reviewed elsewhere [8],[49]-[52]. In brief, a biopsy-sized sample of primary or metastatic tumor is transferred directly into an immunodeficient mouse by orthotopic or subcutaneous implantation. Once tumor engraftment has occurred, RNA and DNA sequencing or chip-based analysis is employed to compare the patient tumor to the PDX. PDXs maintain fidelity to the patient tumor based on molecular subtypes, mutational spectrum, copy number variations, gene expression profiles, and histopathology [50],[53]-[56]. PDX models faithfully recapitulate the intra-tumor heterogeneity and response to chemotherapy [53]. This close resemblance between the PDXs and the patient tumor makes it a suitable predictive preclinical model. The deployment of PDXs therefore can be considered a ‘test bed’ for personalized precision medicine in which genome-forward hypotheses can be assessed preclinically. However, despite the great promise and utility of PDXs, there are some drawbacks that need to be resolved to ensure wider adoption and improved utility. The limitations are the higher comparative cost, high level of technical expertise needed, the lack of an immune system, the effect of differences between the mouse and human microenvironment, and the degree of genetic drift and how this affects conclusions regarding biological and pharmacological findings.

Even with the mentioned limitations, the PDX model has great utility in breast cancer research. Through the genome sequencing of different PDX lines, Li and colleagues [12] identified new ESR1 point mutations and translocations. These gene mutations and the ESR1-YAP1 gene fusion were further investigated through functional studies that directly implicated them in resistance to treatment. Not coincidentally, the patients from whom these PDXs were derived presented with endocrine treatment resistance during their course of treatment.

## Future areas of research

### Proteomics as the next step in the annotation of the breast cancer genome

A fundamental problem in the study of cancer genomics at the level of DNA and RNA is that conclusions regarding pathway activation are indirect since proteins, not nucleic acids, execute these functions. Thus, when signaling and biology are discussed, it is through inference from signal transduction databases that may or may not have been conducted in the relevant biological context and that may or may not be correct. Informatics approaches generate hypotheses, not conclusions [57],[58]. The reverse phase protein array (RPPA) is one answer to the problem of efficiently tracking protein levels and phosphorylation events [59]. Here, tumor protein extracts from many tumors are spotted into slides and probed with highly quality-controlled antibodies. Unfortunately, the generation of RPPA-quality antibodies is technically challenging; in particular, the number of phosphosite-specific antibodies is very limited. Therefore, mass spectrometry is being developed to examine the protein biochemistry of the cancer cells in less biased ways by direct protein sequencing and mass analysis to determine post-translational modifications [3]. Next-generation proteomic technologies are poised to provide deep information on tumor proteomes and on post-translational modifications of all types. When combined with genomic data, proteomics may enable a deeper understanding of complex mechanisms that regulate gene function and dysfunction in cancer. These objectives are being realized by the National Cancer Institute Clinical Proteomic Tumor Analysis Consortium, which is applying standardized proteome analysis platforms to analyze tumor tissues from the TCGA program as well as unique cell and xenograft models and other tissue collections, all of which are accompanied by rich genomic datasets [60].

## Conclusions

The expansion of knowledge in genomics is already having a profound effect on breast cancer research and increasingly on treatment. It is clear, however, that genome-sequencing studies have still not been adequately designed to address specific questions in breast cancer oncology. This is essential to translate the comprehensive catalog of recurrent mutations in breast cancer to a functionally and pharmacologically annotated treatment road map. Through the sequencing of tumors in different time-points, we will be able to identify cellular pathways and targets for drug development and use this information for the development of clinically testable hypotheses. Integrated approaches that not only account for DNA and RNA aberrations but also document protein function and biochemistry are clearly the next technical horizon [60].

## Authors’ contributions

All the authors made substantial contributions to the conception and design of this article, participated in drafting the article or revising it critically for important intellectual content, and gave final approval of the version submitted.

## Authors’ original submitted files for images

Below are the links to the authors’ original submitted files for images. Authors’ original file for figure 1 Authors’ original file for figure 2 Authors’ original file for figure 3

## Abbreviations

AKT1: v-akt murine thymoma viral oncogene homolog1
APOBEC: Apolipoprotein B mRNA editing enzyme, catalytic polypeptide-like
BRCA1: Breast cancer 1, early onset
BRCA2: Breast cancer 2, early onset
CDH1: Cadherin 1, type 1, E-cadherin (epithelial)
ESR1: Estrogen receptor 1
GATA3: GATA binding protein 3
HER2: Human epidermal growth factor receptor 2
*lnc*: Long non-coding
LRP2: Low density lipoprotein receptor-related protein 2
MALAT1: Metastasis associated lung adenocarcinoma transcript 1
MAP3K1: Mitogen-activated protein kinase kinase kinase 1, E3 ubiquitin protein ligase
PDX: Patient-derived xenograft
PIK3CA: Phosphatidylinositol-4,5-bisphosphate 3-kinase, catalytic subunit alpha
RNA seq: RNA sequencing
RPPA: Reverse phase protein array
RYR2: Ryanodine receptor 2 (cardiac)
SMG: Significantly mutated gene
TCGA: The cancer genome Atlas
TP53: Tumor protein p53
