# Supplementary figures and images for: New concepts in breast cancer genomics and genetics
Source: Breast Cancer Res. 2014 Oct 23;16:460. doi: 10.1186/s13058-014-0460-4 (PMC4384360; doi:10.1186/s13058-014-0460-4)

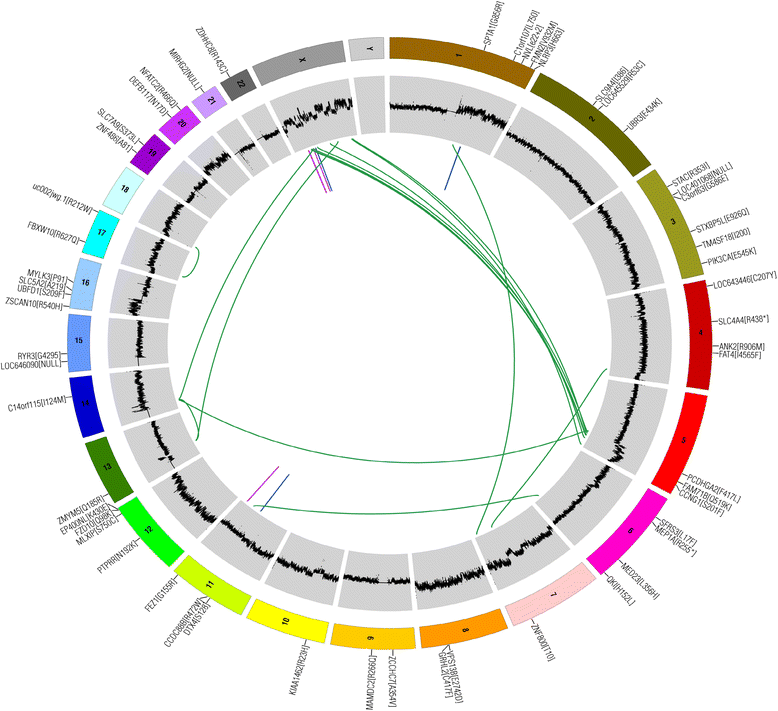

Supplement: Supplementary file 1 — Authors’ original file for figure 1 [file 13058_2014_460_MOESM1_ESM.gif]

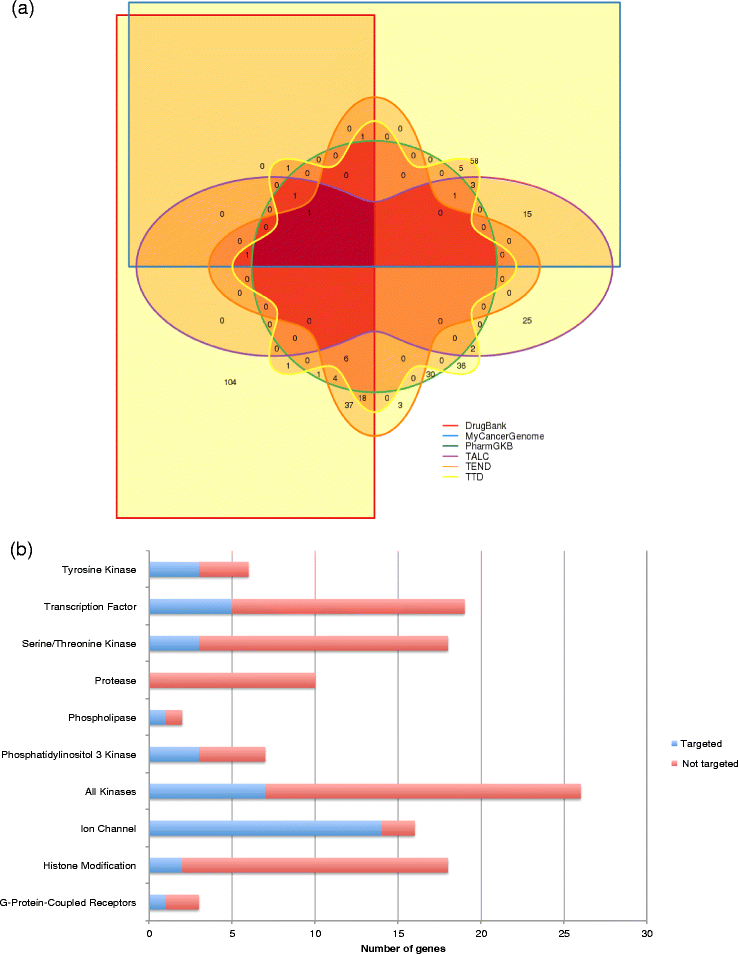

Supplement: Supplementary file 2 — Authors’ original file for figure 2 [file 13058_2014_460_MOESM2_ESM.gif]

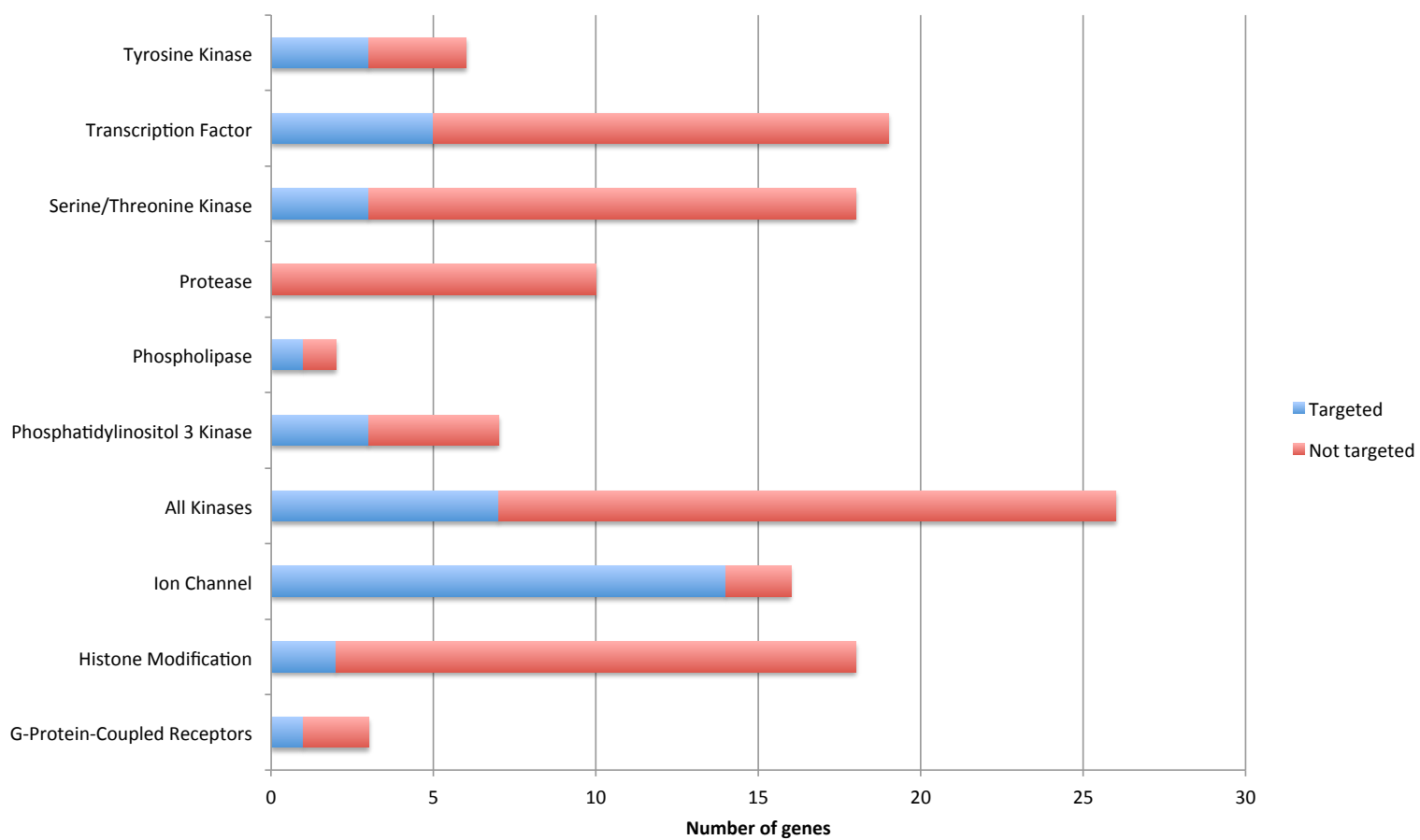

Supplement: Supplementary file 3 — Authors’ original file for figure 3 [file 13058_2014_460_MOESM3_ESM.pdf]
